# Inequities in the Time to Colon Cancer Diagnosis Among Individuals With Severe Psychiatric Illness

**DOI:** 10.1002/cam4.70623

**Published:** 2025-02-17

**Authors:** Jonah H. Gorodensky, Laura Davis, Rebecca Griffiths, Oyedeji Ayonrinde, Colleen Webber, Timothy P. Hanna, Natalie Coburn, Alyson L. Mahar

**Affiliations:** ^1^ Division of Cancer Care and Epidemiology Queen's Cancer Research Institute Kingston Ontario Canada; ^2^ School of Medicine, Queen's University Kingston Ontario Canada; ^3^ ICES Queen's, Queen's University Kingston Ontario Canada; ^4^ Department of Psychiatry Queen's University/Providence Care Hospital Kingston Ontario Canada; ^5^ Ottawa Hospital Research Institute, Bruyère Research Institute Ottawa Ontario Canada; ^6^ Department of Oncology Queen's University Kingston Ontario Canada; ^7^ Department of Surgery, Odette Cancer Centre Sunnybrook Health Sciences Centre Toronto Ontario Canada

**Keywords:** colon cancer, diagnostic delay, diagnostic interval, diagnostic pathways, inequalities, inequities, severe psychiatric illness

## Abstract

**Introduction:**

Early colon cancer detection is critical for improving outcomes. The diagnostic interval is a useful approach to conceptualizing time‐to‐diagnosis within the healthcare system and understanding the diagnostic journey. Adults with severe psychiatric illness (SPI) are less likely to participate in cancer screening and more likely to be diagnosed with advanced cancers. We investigated the association between having an SPI and the colon cancer diagnostic interval.

**Methods:**

We conducted a cross‐sectional study of adults diagnosed with colon cancer in Ontario, Canada between 2007 and 2019 using administrative health data. Individuals with healthcare encounters consistent with pre‐existing major depression, schizophrenia, bipolar disorder, or other non‐organic psychotic illnesses were considered as having SPI. Individuals with an SPI‐related hospitalization were categorized as having an inpatient SPI; the rest were considered outpatient. We calculated the diagnostic interval as the number of days from first colon cancer‐related healthcare encounter to cancer diagnosis. Diagnostic pathways were assessed descriptively, including whether diagnosis was made symptomatically or with no symptom recorded. Quantile regression (stratified by symptom status) was used to quantify the association between SPI status and the diagnostic interval.

**Results:**

We identified 42,143 individuals with colon cancer: 40,884 with no history of mental illness, 835 with a history of outpatient SPI, and 424 with inpatient SPI. Adults with SPI were significantly more likely to be diagnosed symptomatically (inpatient: 89.9%, outpatient: 86.6%, no SPI: 80.9%, *p* < 0.001). Individuals with SPI experienced a significantly longer median symptomatic diagnostic interval and a similar median diagnostic interval when diagnosed with no symptom recorded, relative to those without a history of mental illness. After adjusting for covariates, the median symptomatic diagnostic interval was 48 days longer (95% CI 28, 68) among individuals with outpatient SPI and 55 days longer (95% CI 28, 82) among individuals with inpatient SPI compared to those with no SPI.

**Conclusion:**

Individuals with SPI were more likely to be diagnosed symptomatically and had longer symptomatic diagnostic intervals than those without. This study represents a first step in targeting and improving cancer diagnostic processes for individuals with SPI.

## Abstract 2

Our study demonstrates that individuals with severe psychiatric illness are more likely to be diagnosed with colon cancer symptomatically and experience longer time to diagnosis.

## 1 Introduction

Adults with severe psychiatric illness (SPI) experience worse cancer outcomes and a higher‐than‐expected cancer case‐fatality rate, across a variety of cancers [1, 2, 3, 4, 5, 6, 7, 8]. This is in spite of individuals with SPI having a similar cancer incidence compared to the rest of the population [1, 2, 3]. Looking at colon cancer in particular, individuals with SPI are less likely to be screened, more likely to be diagnosed with advanced or metastatic stage cancer, experience non‐guideline‐recommended treatment patterns and have worse survival, while the incidence of colon cancer itself is not increased [3, 4, 5, 6, 9, 10]. However, areas for mitigating these outcomes within the healthcare system is unclear, with differences previously identified in both the diagnostic and treatment patterns of individuals with SPI [2, 6, 11, 12, 13].

There have been several proposed mechanisms for differential cancer outcomes in individuals with SPI and the causes are likely multifactorial. Acknowledging that the underlying SPI impacts how an individual interacts with the healthcare system, systems‐level mechanisms can more readily be targeted to try and rectify differential cancer outcomes. Diagnostic overshadowing, the process by which unrelated physical issues are attributed by health professionals to a pre‐existing mental illness rather than physical illness, is an oft‐cited cause of differential outcomes in individuals with SPI [1, 2, 14, 15]. The siloing of physical and mental healthcare resources can also contribute to the piecemeal administration of healthcare to individuals with SPI, leading routine preventative healthcare and screening to be missed [1, 2, 9, 15]. Furthermore, individuals with SPI are more likely to be lower income, and face significant stigmatization, both of which lead to poorer care [16].

Colon cancer is a major cause of morbidity and mortality worldwide, and early detection, ideally through screening, is understood to be critical for improving overall survival rates [17]. Measuring the diagnostic interval can be used as a proxy for measuring delays in cancer diagnosis and emerging evidence suggests that there is significantly longer colon cancer diagnostic interval in individuals with SPI [11]. This emerging evidence, however, groups together multiple cancer sites and controls for variables on the causal pathway making it difficult to identify concrete areas amenable to systems‐level interventions to address these disparities. Therefore, in this study, we aim to: (1) describe the ways in which individuals with SPI are diagnosed with colon cancer and (2) investigate the association between having an SPI and the length of the colon cancer diagnostic interval, while considering potential confounders and causal pathway variables.

## 2 Methods

### 2.1 Study Population

We conducted a cross‐sectional study using linked administrative health databases in Ontario, Canada. Ontario has a population of over 15.5 million, nearly all of whom are eligible for publicly funded health care. Ontario has a robust colon cancer screening program which is free to access [18]. Mental health care by psychiatrists is included in the publicly funded system and typically requires a referral from another physician to access. All adults with a first colon cancer diagnosis (International Classification of Diseases for Oncology (ICD‐O‐3) codes C18.0, C18.2–C18.9) between January 1, 2007 and December 31st, 2019 were included. We excluded individuals who were < 18 years old at colon cancer diagnosis, had < 2 years of public Ontario Health Insurance Plan (OHIP) coverage prior to colon cancer diagnosis, had an invalid death date or the diagnosis appeared only on a death certificate, had a history of any other malignancy or were diagnosed with another malignancy on the date of colon cancer diagnosis, or had no identifiable first contact date (the earliest recorded healthcare encounter related to a colon cancer diagnosis) with the health system. The study received ethical clearance from the Queen's University Faculty of Health Sciences and Affiliated Hospitals Research Ethics Board (#NURS‐559‐22).

### 2.2 Data Sources

Data were housed and analyzed at ICES (formerly the Institute for Clinical Evaluative Sciences), and individuals were linked using unique encoded identifiers. ICES is an independent, non‐profit research institute whose legal status under Ontario's health information privacy law allows it to collect and analyze health care and demographic data, without consent, for health system evaluation and improvement. The following databases were used: the Canadian Institute for Health Information (CIHI) Discharge Abstract Database (DAD) (CIHI‐DAD) and the Ontario Mental Health Reporting System (OMHRS), which contain all records from psychiatric hospital admissions in Ontario; the OHIP database and ICES physician database, which contain physician billing and physician specialty information; the National Ambulatory Care Reporting System (NACRS), which includes information on emergency department visits; the Ontario Cancer Registry (OCR); and the Registered Persons Database (RPDB), which includes demographic information and vital status.

### 2.3 Study Variables

#### 2.3.1 Measuring Severe Psychiatric Illness

We assessed SPI status in the 5 years to 6 months prior to the cancer diagnosis using an established algorithm based on hospitalization data, emergency department visits, and physician visits for major depression, schizophrenia, bipolar disorder, and other non‐organic psychotic illnesses (Table S1) [4, 6, 19]. A history of SPI was hierarchically classified as being inpatient (≥ 1 hospitalization for an eligible diagnosis) or outpatient (≥ 2 emergency department or psychiatrist visits for an eligible diagnosis) as a proxy for the severity of their mental health condition [4, 7, 20]. Individuals with a history of mental health encounters in the time frame that did not meet our definition of SPI (e.g., non‐SPI diagnoses only, single ED visit, single psychiatrist visit, encounters with family physicians only) were excluded from the study. The relationship between individuals with *less severe* psychiatric illness and the diagnostic interval is likely complex and dissimilar to that of individuals with SPI; as such, and in keeping with previously published works using ICES data, we have excluded this cohort [4, 6, 19].

#### 2.3.2 Outcomes

The main outcome of interest was the diagnostic interval length, which, following the Aarhus statements [21], is defined as the number of days from the earliest healthcare encounter related to colon cancer to the diagnosis date. The diagnostic interval was calculated using a previously established method [22, 23, 24, 25]. Briefly, we identified all physician claims, hospital discharges, and emergency department visits to identify and categorize encounters and diagnoses which occurred at least 20% more frequently in the 0–3 months prior to colon cancer diagnosis as compared to a control period 24–27 months before diagnosis. Similar encounter types were grouped together and, using statistical control charts, we identified appropriate look‐back periods for each encounter to allow us to identify the first contact date for each patient. The first contact date was the earliest relevant healthcare encounter for a relevant encounter type. If the first contact date was a procedure, we looked back an additional 365 days to identify a previous visit with the referring physician. The diagnostic interval was then calculated as the number of days between the first contact date (or referring physician date) and the colon cancer diagnosis date.

In addition to the diagnostic interval, we also looked at several other variables pertaining to how the colon cancer was diagnosed, based on recommended colon cancer diagnostic pathways [25, 26, 27]. We categorized patients as being diagnosed with no symptom recorded or symptomatically based on the first contact date. The no symptom recorded group was defined in the administrative data as individuals who received a non‐emergent guaiac fecal occult blood test (gFOBT) or lower gastrointestinal (GI) scope (including colonoscopy) on the first contact date without co‐recorded symptoms and had no previously recorded symptoms. Screening for colon cancer is not recorded in ICES data, but these tests, in the absence of previously recorded symptoms, would theoretically represent the colon cancer screening pathways in Ontario [27]. Emergent diagnoses were defined as an emergency department visit on the date of diagnosis. Any individual who did not meet this definition was considered as having a symptomatic diagnostic pathway.

We further subcategorized the diagnostic pathway among individuals diagnosed symptomatically. We assessed all procedures between the first contact date and colon cancer diagnosis, and pathways were assigned as one of four pathways: colonoscopy alone, colonoscopy and another imaging modality, imaging only, and neither imaging nor colonoscopy [26]. All diagnostic pathways were further subdivided based on whether the cancer was diagnosed emergently, for a total of eight pathways.

#### 2.3.3 Demographic and Cancer Variables

We assessed demographic and cancer‐related variables during the 12 months prior to the colon cancer diagnosis with the exception of comorbidities, which were measured in the 2 years prior using the Elixhauser comorbidity index. The Elixhauser comorbidity index is a commonly used method of quantifying comorbidity burden in administrative health databases. Elixhauser scores were reported dichotomized to < 4 (less comorbid) and ≥ 4 (more comorbid) in our study [28]. Age at cancer diagnosis and sex (female/male) were measured from the RPDB. Individual or family income was unavailable in our data sources; income was measured through postal codes linked to census‐based neighborhood income quintiles as a proxy. Postal codes were also used to assess rurality of a persons' residence using the Rurality Index of Ontario (RIO), which provides a score attempting to quantify how rural a community is as a function of population size, distance to family practitioners, and travel time to access healthcare [29]. RIO values were dichotomized to being < 45 (urban) or ≥ 45 (rural). Colon cancer characteristics included year of diagnosis, tumor type (adenocarcinoma or other), and stage at diagnosis.

### 2.4 Statistical Analysis

We described demographic and diagnostic pathway variables as counts and proportions for categorical variables and means, medians, and interquartile ranges for continuous variables. Univariable and multivariable quantile regression were used to estimate the association between SPI status and the diagnostic interval length, with inpatient and outpatient SPI considered separately (reference group no history of mental illness). Quantile regression is a well‐established method of analyzing skewed data and allows for modeling of data at different centiles. These models allow us to account for the right‐skewed nature of diagnostic intervals [30]. Due to conceptually different healthcare pathways for individuals diagnosed with no symptom recorded as compared to those diagnosed symptomatically, all analyses were a priori stratified by symptom status at diagnosis. Effect estimates are presented at the 50th (median) and 90th percentiles with their respective 95% confidence intervals (CI). We also investigated an interaction between the diagnostic interval length and sex to evaluate the effect of an SPI within females and males separately. Sex was chosen as a potential interaction due to its well‐understood relationship with prolonged diagnostic intervals [31] and the sex differences in the distribution among certain severe psychiatric illnesses (i.e., depression is more common in females [32], schizophrenia is less [33]).

Multivariable quantile regressions were adjusted for age (continuous), sex (binary), rurality, and year of colon cancer diagnosis. We selected confounders using the principles of effect decomposition for health equity research outlined by Jackson [34]. This framework suggests considering both whether a variable can be considered a confounder by traditional casual inference and whether the distribution of the variable with respect to the exposure of interest is unfair. We believe that pre‐existing comorbidities and income are likely on the causal pathway between having a SPI and the diagnostic interval and represent an unfair discrepancy between individuals with and without an SPI, so were not included as confounders. SAS version 9.4 was used for all analyses, and figures were generated using R version 3.6.1 [35, 36].

## 3 Results

### 3.1 Study Cohort

**FIGURE 1:**
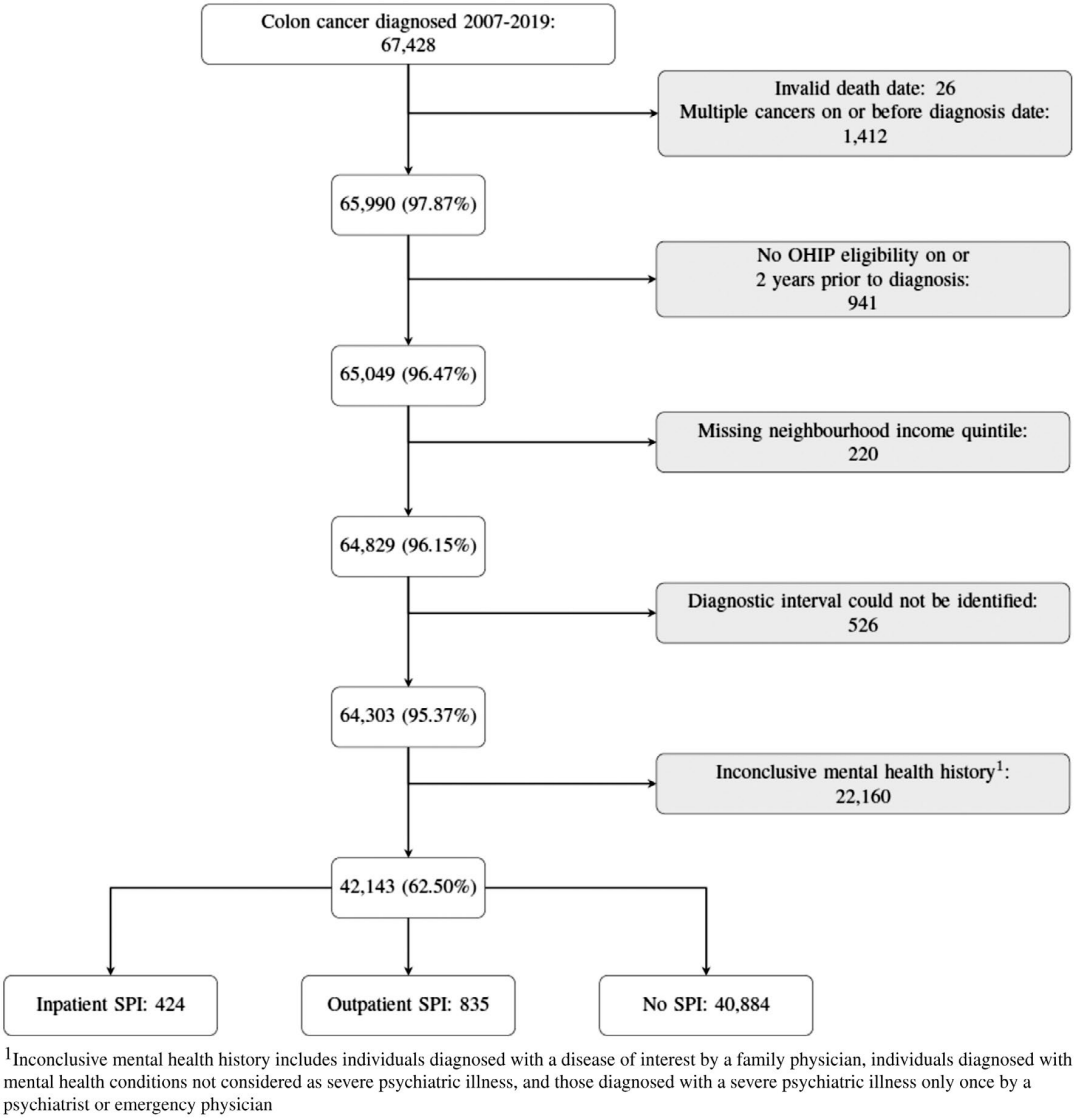
Cohort inclusions and exclusions. Inconclusive mental health history includes individuals diagnosed with a disease of interest by a family physician, individuals diagnosed with mental health conditions not considered as severe psychiatric illness, and those diagnosed with a severe psychiatric illness only once by a psychiatrist or emergency physician.

This study included 42,143 persons with colon cancer, of whom 40,884 had no history of SPI, 835 had a history of an outpatient SPI, and 424 had an inpatient SPI (Figure 1). Table 1 presents the demographic and colon cancer characteristics of this cohort. Individuals with an SPI were diagnosed at a younger age (median [IQR]: inpatient SPI: 67 [58–76], outpatient: 67 [58–76], no SPI: 72 [62–80], *p* < 0.001), were more likely to be female (inpatient SPI: 52.4%, outpatient SPI: 53.7%, no SPI: 44.7%; *p* =< 0.001), lived in neighborhoods in lower income quintiles (1st income quintile; inpatient SPI: 33.7%, outpatient SPI: 28.0%, no SPI: 19.7%; *p* =< 0.001), and experienced a higher comorbidity burden (Elixhauser ≥ 4; inpatient SPI: 27.1%, outpatient SPI: 21.4%, no SPI: 12.6%; *p* < 0.001). There were no statistically significant differences in the distribution of year of colon cancer diagnosis (*p* = 0.47), stage at diagnosis (*p* = 0.07), or type of colon cancer (adenocarcinoma versus another tumor type, *p* = 0.93) among the groups.

**TABLE 1:** Demographic and colon cancer characteristics by severe psychiatric illness status, *n* (%).

	History of SPI, *N* = 1258		No SPI	*p* ^a^
	Inpatient SPI	Outpatient SPI		
	*N* = 424	*N* = 835	*N* = 40,884	
Age at diagnosis				
≤ 50	44 (10.4%)	79 (9.5%)	2741 (6.7%)	< 0.001
51–60	97 (22.9%)	183 (21.9%)	5743 (14.0%)	
61–70	125 (29.5%)	251 (30.1%)	10,191 (24.9%)	
71–80	101 (23.8%)	192 (23.0%)	12,297 (30.1%)	
> 80	57 (13.4%)	130 (15.6%)	9912 (24.2%)	
Sex				
Female	222 (52.4%)	448 (53.7%)	18,292 (44.7%)	< 0.001
Rural residence				
RIO ≥ 45	35 (8.3%)	27 (3.2%)	3170 (7.8%)	< 0.001
Elixhauser comorbidity burden				
< 4	309 (72.9%)	656 (78.6%)	35,737 (87.4%)	< 0.001
≥ 4	115 (27.1%)	179 (21.4%)	5147 (12.6%)	
Neighborhood income quintile				
1	143 (33.7%)	234 (28.0%)	8057 (19.7%)	< 0.001
2	75 (17.7%)	169 (20.2%)	8544 (20.9%)	
3	73 (17.2%)	152 (18.2%)	8234 (20.1%)	
4	75 (17.7%)	142 (17.0%)	7978 (19.5%)	
5	58 (13.7%)	138 (16.5%)	8071 (19.7%)	
Year of diagnosis				
2007	36 (8.5%)	56 (6.7%)	3006 (7.4%)	0.472
2008	40 (9.4%)	69 (8.3%)	3048 (7.5%)	
2009	22 (5.2%)	69 (8.3%)	3082 (7.5%)	
2010	43 (10.1%)	62 (7.4%)	3064 (7.5%)	
2011	29 (6.8%)	60 (7.2%)	3199 (7.8%)	
2012	33 (7.8%)	61 (7.3%)	3140 (7.7%)	
2013	29 (6.8%)	60 (7.2%)	3139 (7.7%)	
2014	25 (5.9%)	61 (7.3%)	3121 (7.6%)	
2015	39 (9.2%)	79 (9.5%)	3194 (7.8%)	
2016	32 (7.5%)	61 (7.3%)	3214 (7.9%)	
2017	42 (9.9%)	72 (8.6%)	3307 (8.1%)	
2018	28 (6.6%)	63 (7.5%)	3165 (7.7%)	
2019	26 (6.1%)	62 (7.4%)	3205 (7.8%)	
Histology				
Adenocarcenoma	412 (97.2%)	809 (96.9%)	39,701 (97.1%)	0.929
Stage at diagnosis				
I	65 (15.3%)	146 (17.5%)	7564 (18.5%)	0.066
II	87 (20.5%)	209 (25.0%)	10,246 (25.1%)	
III	116 (27.4%)	190 (22.8%)	9935 (24.3%)	
IV	94 (22.2%)	158 (18.9%)	7366 (18.0%)	
Unknown/missing	62 (14.6%)	132 (15.8%)	5773 (14.1%)	

Abbreviation: RIO, rurality index of Ontario.

^a^ *p* value calculated using the chi‐square test.

### 3.2 Description of the Diagnostic Pathway

Table 2 presents the symptom status and pathways of diagnosis among the three cohorts. Individuals with an SPI were significantly less likely to be diagnosed with colon cancer with no symptom recorded as compared to individuals without such a history (inpatient SPI: 10.1%, outpatient SPI: 13.4%, no SPI: 19.1%; *p* =< 0.001). Among individuals diagnosed symptomatically, those with inpatient SPI were more likely to visit an emergency department on the date of diagnosis (44.9%) as compared to individuals with an outpatient SPI (33.6%) or no SPI (33.5%).

**TABLE 2:** Cohorts by symptom status and pathways of diagnosis.

	History of SPI, *N* = 1258		No SPI	*p* ^a^
	Inpatient SPI	Outpatient SPI		
	*N* = 424	*N* = 835	*N* = 40,884	
No symptom recorded pathway	43 (10.1%)	112 (13.4%)	7791 (19.1%)	< 0.001^b^
Diagnostic interval, median (IQR), 90 percentile	62 (35, 145), 198	67 (34, 122), 204	69 (34, 134), 222	0.723
Symptomatic pathway	381 (89.86%)	723 (86.59%)	33,093 (80.9%)	< 0.001^b^
Diagnostic interval, median (IQR), 90 percentile	160 (44, 313), 438	147 (44, 310), 433	106 (23, 244), 387	< 0.001
Diagnostic pathway summary				
Colonoscopy only + ED	23 (6.0%)	33 (4.6%)	1594 (4.8%)	< 0.001
Colonoscopy only + no ED	72 (18.9%)	170 (23.5%)	9410 (28.4%)	
Colonoscopy + imaging + ED	61 (16.0%)	90 (12.4%)	3993 (12.1%)	
Colonoscopy + imaging + no ED	66 (17.3%)	138 (19.1%)	5617 (17.0%)	
Imaging only + ED	79 (20.7%)	108 (14.9%)	5007 (15.1%)	
Imaging only + no ED	58 (15.2%)	126 (17.4%)	4648 (14.0%)	
No imaging, no colonoscopy + ED	8 (2.1%)	12 (1.7%)	474 (1.4%)	
No imaging, no colonoscopy + no ED	14 (3.7%)	46 (6.4%)	2350 (7.1%)	
ED visit on index diagnosis date	171 (44.9%)	243 (33.6%)	11,068 (33.4%)	

Abbreviations: ED, emergency department; IQR, interquartile range; SPI, severe psychiatric illness.

^a^ *p* value calculated using the chi‐square test except for rows displaying diagnostic intervals, which are calculated using the Kruskal–Wallis test.

^b^ This value represents the chi‐square test for the proportions between the no‐symptom‐recorded and symptomatic pathways.

### 3.3 Diagnostic Intervals

**FIGURE 2:**
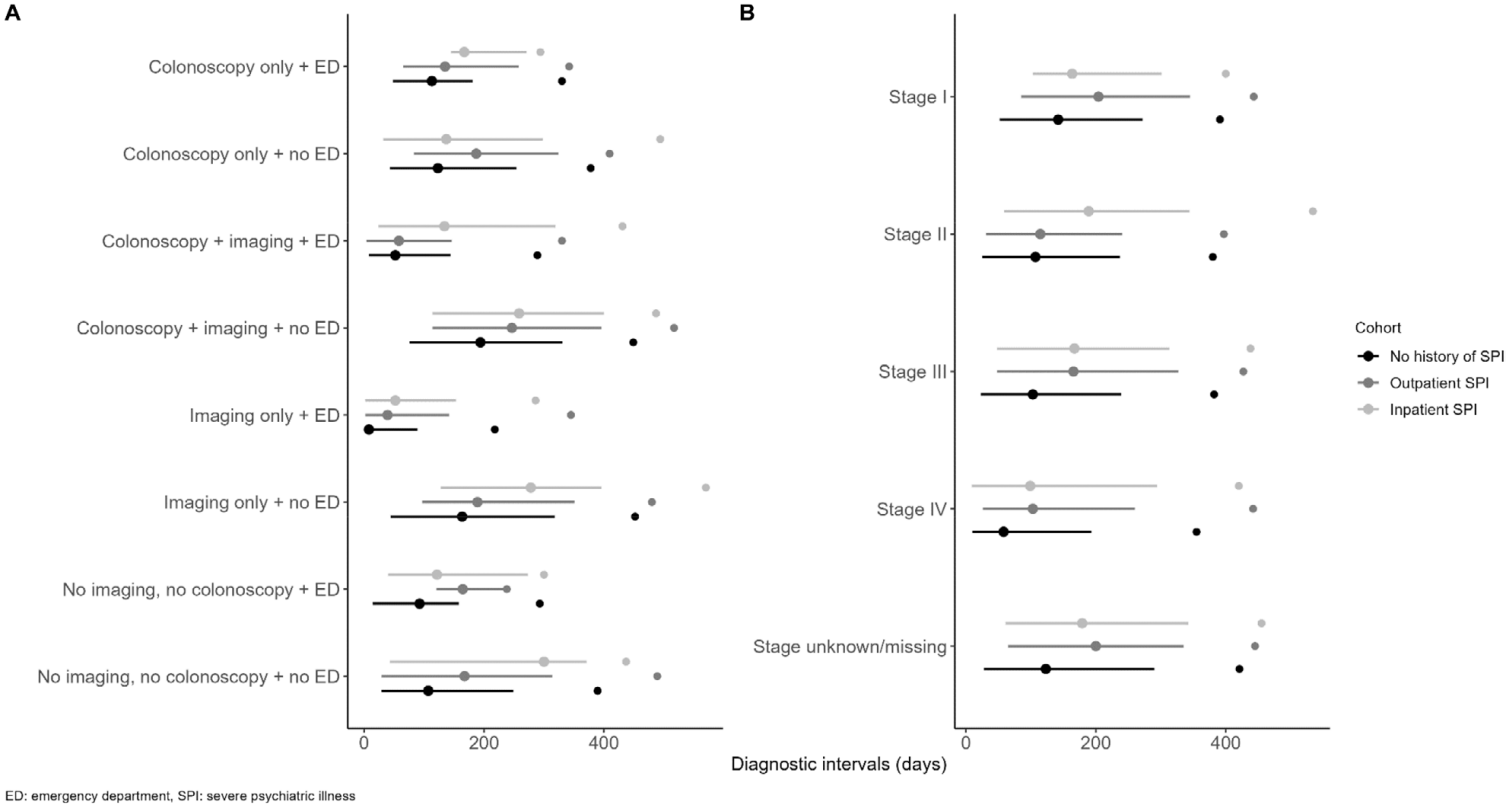
Median and interquartile range diagnostic intervals for (A) diagnostic pathways and (B) stage at diagnosis among patients diagnosed symptomatically. Points on the far right represent the 90th percentile. Points with lines represent the 50th percentile, with the line representing the 25th and 75th percentiles.

Among individuals diagnosed symptomatically, across all eight assessed diagnostic pathways individuals with SPI had consistently longer median diagnostic intervals than patients with no SPI (see Figure 2). For example, when the diagnosis was made non‐emergently via colonoscopy and imaging, individuals with an inpatient SPI (median: 259 IQR: 114, 400) or outpatient SPI (median: 247 IQR: 114, 396) had median diagnostic intervals 65 and 53 days longer than individuals with no SPI (median: 194 IQR: 76, 331) respectively. As well, at each cancer stage at diagnosis (including missing/unknown) individuals with SPI had a significantly longer diagnostic interval than individuals with no SPI.

### 3.4 Quantile Regression

Table 3 presents the results of the unadjusted and adjusted quantile regressions at the 50th and 90th percentiles, stratified by symptom status at diagnosis. Among individuals in the no symptom recorded group, there were no differences in the length of the diagnostic interval in either unadjusted or adjusted models between those with and without SPI. Within the cohort of individuals diagnosed symptomatically, however, effect estimates demonstrated a consistently prolonged diagnostic interval in individuals with SPI compared to those without. For example, at the 50th percentile, after adjusting for covariates, patients with an outpatient SPI had a diagnostic interval 48 (95% CI 28, 68) days longer than patients with no SPI, and patients with an inpatient SPI had a diagnostic interval 55 (95% CI 28, 82) days longer. There was no significant effect of adding sex as an interaction term with SPI (Figure S1).

**TABLE 3:** Quantile regression for the effect of SPI on the diagnostic interval.

	Unadjusted	Adjusted
	Estimate (95% CI)	Estimate (95% CI)
No symptom recorded		
50th percentile		
Intercept	69.0 (66.7, 71.3)	38.3 (22.9, 53.6)
Inpatient	−7.0 (−25.8, 11.8)	−2.0 (−30.6, 24.6)
Outpatient	−1.6 (−16.3, 13.1)	−3.3 (−17.0, 10.3)
90th percentile		
Intercept	222.0 (214.8, 229.2)	125.8 (102.0, 149.6)
Inpatient	−24.0 (−85.3, 37.3)	−40.4 (−91.0, 20.3)
Outpatient	−18.0 (−93.8, 57.8)	−8.9 (−55.0, 37.3)
Symptomatic		
50th percentile		
Intercept	106.0 (104.2, 107.8)	10.8 (−4.1, 25.8)
Inpatient	54.0 (33.4, 74.6)	54.7 (27.7, 81.7)
Outpatient	41.0 (18.4, 63.6)	48.1 (27.8, 68.4)
90th percentile		
Intercept	387.0 (383.5, 390.5)	281.3 (261.1, 301.6)
Inpatient	51.0 (6.8, 95.2)	64.9 (33.2, 96.7)
Outpatient	46.0 (24.2, 67.9)	54.7 (29.4, 79.9)

*Note:* Multivariable regressions were adjusted for age, sex, rurality, and year of diagnosis. Values labeled intercept represent the diagnostic interval at baseline for those without SPI, of median age, male sex, living in a rural area and diagnosed in 2019. Values labeled inpatient or outpatient represent the additional diagnostic interval length for an individual with inpatient or outpatient SPI. Positive values indicate longer diagnostic intervals, negative values indicate shorter intervals.

Abbreviations: CI, confidence interval; SPI, severe psychiatric illness.

## 4 Discussion

In our large, population‐based study of diagnostic pathways and the diagnostic interval among colon cancer patients in Ontario, Canada, we showed that individuals with an SPI experienced consistently worse diagnostic outcomes than individuals without. First, individuals with SPI were significantly more likely to be diagnosed symptomatically compared to those with no history of mental illness. Although our definition of colon cancer detection without a recorded symptom does not reveal the reason a test was ordered or rule out the presence of symptoms, we believe that detection without a symptom recorded likely serves as a reasonable surrogate for colon cancer screening. In support of this, our rate of colon cancer diagnosis with no symptom recorded in the no SPI cohort (19%) closely reflects the rates of diagnosis via true screening in other studies [37, 38]. It should be noted that individuals with SPI were diagnosed younger than individuals without SPI, though the vast majority of individuals with SPI were diagnosed after age fifty and should thus have still been eligible for provincial screening, regardless of family history. It is well known that individuals with SPI are less likely to be screened than individuals without for other cancer sites (i.e., breast, cervical); our result, though unable to quantify true screening, adds to the small but growing body of evidence that there is also differential colon cancer screening [1, 2, 10, 39, 40]. Expanding on this finding, we show that, among individuals with SPI and no recorded symptom, diagnostic intervals were no longer than individuals without SPI diagnosed with no recorded symptom, evidence to the importance of ensuring this population is given adequate access to screening and support to engage in the screening process [27].

We also demonstrated that among individuals diagnosed symptomatically, those with SPI experienced significantly longer diagnostic intervals than those without SPI—more than 7 weeks longer in individuals with SPI as compared to individuals without SPI based on 50th percentile quantile regression. Coupled with the fact that individuals with SPI were more likely to be diagnosed symptomatically, our findings point to a significant inequity. Benitez Majano et al. also looked at time to diagnosis in colon cancer in England and showed that patients with any psychiatric illness experienced diagnostic intervals nearly twice as long as patients without [11]. Though their definitions of psychiatric illness and the cancer diagnosis date differed from ours, and direct comparisons of our diagnostic intervals are therefore difficult to make, the trend towards significantly prolonged diagnostic intervals in patients with psychiatric illness is consistent [11]. In addition to individuals with SPI, other marginalized groups have been shown to experience prolonged diagnostic intervals across a variety of cancers, including living in a neighborhood with low socioeconomic status [41], women [31], immigrants [42], and living in rural areas [22, 43]. The diagnostic intervals were prolonged on the scale of days for these other folks, rather than the weeks or months seen in individuals with SPI observed in our study.

Various interventions have been suggested to try and rectify inequities such as delayed diagnostic intervals, though there are few evidence‐based strategies. One possible intervention would be to minimize gaps between primary care, mental and physical health professionals, such as implementing shared electronic health records, to help care providers stay more aware of the physical and psychiatric issues at play in their patients. Teams could work together to ensure eligible persons were offered adapted or modified cancer screening protocols and increase opportunities for atypical results to be addressed with subsequent and timely investigation. This could ideally increase screening rates and decrease the time to diagnosis [2]. In this same vein, mental health professionals could play an active collaborative role in ensuring their patients are accessing appropriate cancer screening [15], particularly in the absence of a primary care physician. Patient navigation and peer support services are effective in increasing cancer screening in other marginalized populations and should be considered in patients with SPI, though no studies have suggested that these programs decrease the diagnostic interval [44, 45]. It is also possible that illness severity in SPI affects individual's ability to seek help or follow screening protocols; therefore, interventions that improve and sustain mental wellness more broadly may also improve cancer care outcomes.

Our study has some limitations. First, though our method for calculating the diagnostic interval has been used in several other studies, it has not been validated due to difficulties linking data [22, 23, 24, 25]. As well, by excluding patients diagnosed with colon cancer posthumously, we excluded patients who died while being worked up for colon cancer (though there were few such patients). Patients with SPI may experience slower and/or more fragmented workups and thus may be differentially excluded from our cohort. Due to limitations in our data, we were also unable to look at specific mental health diagnoses, but rather only SPI as a heterogeneous grouping. There may be differences in diagnostic intervals and processes between different underlying diagnoses (i.e., schizophrenia vs. bipolar disorders vs. major depression) which have different symptomology but significant illness burden, but we were unable to investigate this in our study. In addition, although our method has been used by other works [25, 46], we cannot know if patients were truly asymptomatic at diagnosis. By defining diagnosis with no symptom recorded using administrative data that do not contain information on colon cancer screening explicitly, we may have misclassified certain symptomatic individuals whose symptoms were simply not recorded in the data. This said, if anything, individuals with SPI may be less likely to have their symptoms recorded before a colonoscopy/gFOBT as, due to healthcare provider bias, their symptoms may be attributed to their mental health; if this is the case, the true difference may be larger than we have estimated. Finally, our method of calculating the diagnostic intervals relied on identifying the first cancer‐related contact date. It is possible there was differential misclassification of start‐dates between our cohorts of interest, especially if those with SPI were more likely to have early symptoms misattributed or considered psychosomatic. This said, if this was the case, the diagnostic interval would likely be shortened for individuals with SPI, and thus the interpretation of our findings would not change.

Our study demonstrates that even within a publicly funded healthcare system, individuals with SPI experience significant inequities in their diagnosis of colon cancer; they are more likely to be diagnosed symptomatically, more likely to be diagnosed emergently, and experience diagnostic intervals up to 7 weeks longer compared to those without SPI when diagnosed symptomatically. Our findings highlight a gap in cancer care that cannot be explained by biology alone. A prolonged diagnostic interval highlights that differences in cancer care for individuals with SPI begin even before the cancer is diagnosed and may be one of the many reasons individuals with SPI experience inequitable cancer‐related outcomes. Future work should investigate mechanisms by which these differences can be mitigated with the goal of minimizing disparities and ensuring patients with SPI attain equitable cancer outcomes.

## Author Contributions

**Jonah H. Gorodensky:** conceptualization (equal), formal analysis (equal), investigation (equal), methodology (equal), visualization (lead), writing – original draft (lead). **Laura Davis:** conceptualization (equal), data curation (equal), methodology (equal), software (equal), writing – review and editing (equal). **Rebecca Griffiths:** data curation (equal), methodology (equal), software (equal), writing – review and editing (equal). **Oyedeji Ayonrinde:** conceptualization (equal), investigation (equal), writing – review and editing (equal). **Colleen Webber:** methodology (equal), resources (equal), writing – review and editing (equal). **Timothy P. Hanna:** conceptualization (equal), funding acquisition (equal), methodology (equal), writing – review and editing (equal). **Natalie Coburn:** funding acquisition (equal), writing – review and editing (equal). **Alyson L. Mahar:** conceptualization (equal), funding acquisition (equal), investigation (equal), methodology (equal), project administration (equal), supervision (lead), writing – review and editing (equal).

## Conflicts of Interest

The authors declare no conflicts of interest.

## Supporting information


**Table S1.** Diagnosis codes and data sources used to identify relevant healthcare encounters related to a mental illness in the 6 months to 5 years prior to the colorectal cancer diagnosis.
**Figure S1.** Differences in effect estimates after adding interaction term for sex. Values reported represent the effect estimate and 95% confidence intervals.

## Data Availability

The data set from this study is held securely in coded form at ICES. Although data sharing agreements prohibit ICES from making the data set publicly available, access may be granted to those who meet prespecified criteria for confidential access, available at www.ices.on.ca/DAS. The full data set creation plan and underlying analytic code are available from the authors on request, understanding that the programs may rely on coding templates or macros that are unique to ICES.
