# Supplementary material for: Inequities in the Time to Colon Cancer Diagnosis Among Individuals With Severe Psychiatric Illness
Source: Cancer Med. 2025 Feb 17;14(4):e70623. doi: 10.1002/cam4.70623 (PMC11831619; doi:10.1002/cam4.70623)
Supplement: Supplementary file 1 — Table S1. Diagnosis codes and data sources used to identify relevant healthcare encounters related to a mental illness in the 6 months to 5 years prior to the colorectal cancer diagnosis. Figure S1. Differences in effect estimates after adding interaction term for sex. Values reported represent the effect estimate and 95% confidence intervals. [file CAM4-14-e70623-s001.docx]

**Table S1. Diagnosis codes and data sources used to identify relevant healthcare encounters related to a mental illness in the 6 months to five years prior to the colorectal cancer diagnosis**

| Database | Diagnostic Code | *Description* |
| --- | --- | --- |
| OHIP^1^ |  |  |
|  | 295 | Schizophrenia |
|  | 296 | Manic Depressive Psychosis, Melancholia Involutional |
|  | 297  298 | Paranoid states  Other psychoses |
|  | 311 | Depressive or Other Non-psychotic Disorder,  Not Classified Elsewhere |
| CIHI-DAD & NACRS^2^ | |  |
|  | F20–F29 | Schizophrenia, schizotypal and delusional disorders |
|  | F30 -F39 | Mood [affective] disorders |
| OMHRS^3^ |  |  |
|  |  | Mood Disorder  Schizophrenia  Other Psychotic Disorder |

^1^Based on ICD-9; ^2^ICD-10 CA; ^3^DSM-IV; OHIP= Ontario Health Insurance Plan; CIHI-DAD= Canadian Institute of Health Information-Discharge Abstract Database; NACRS=National Ambulatory Reporting System; OMHRS=Ontario Mental Health Reporting System


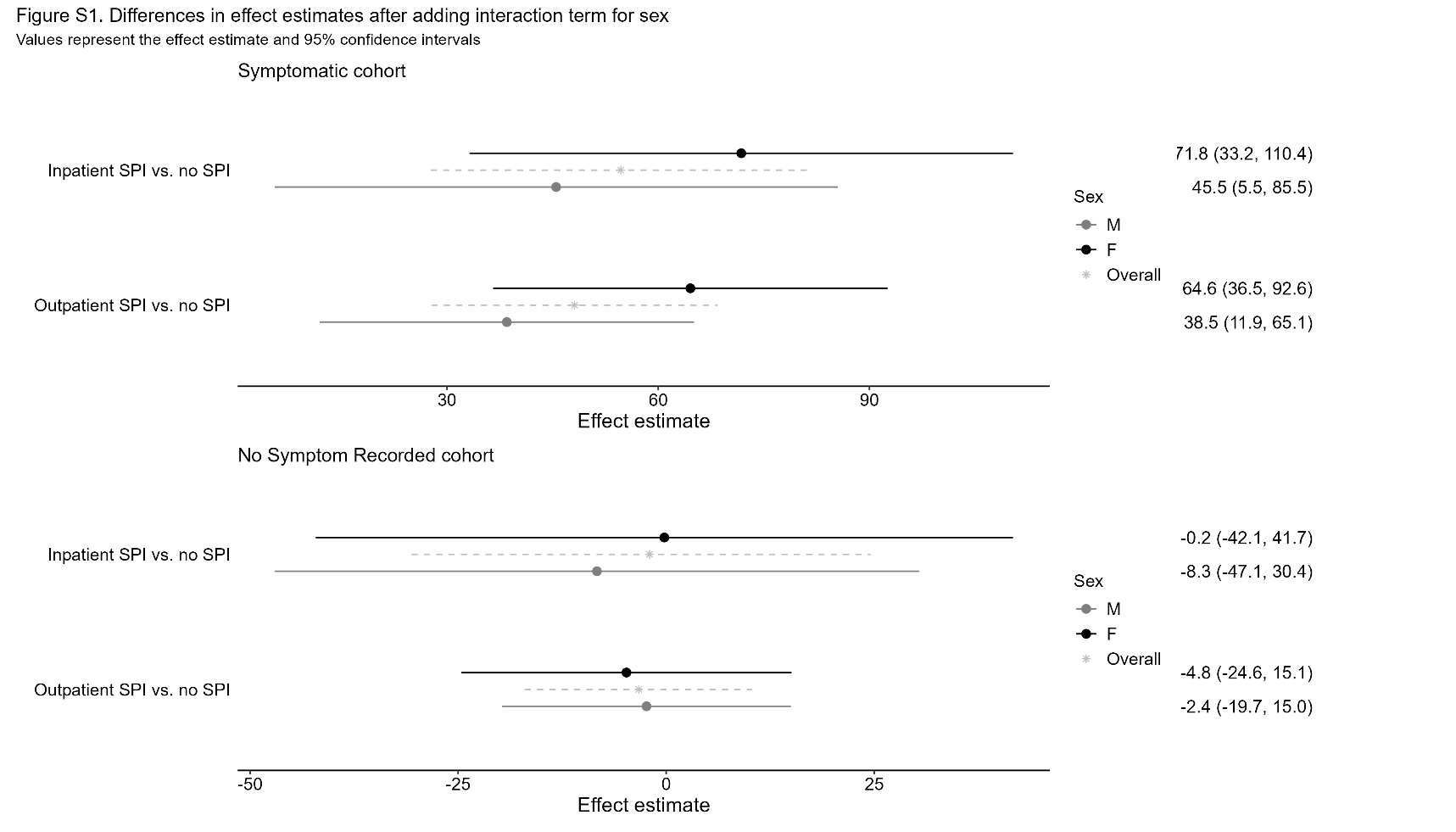


F: female, M: male, SPI: severe psychiatric illness

The dotted line represents the averaged effect estimate for male and females combined.
